# Diagnostic Performance of the Uric Acid‐To‐Albumin Ratio in Predicting Severity of Acute Pancreatitis

**DOI:** 10.1002/jgh3.70471

**Published:** 2026-09-21

**Authors:** Hüsnü Serdar Kızıltunç, Şirin Çetin, Mehmet Batuhan Duman

**Affiliations:** ^1^ Department of Internal Medicine Amasya University Faculty of Medicine Amasya Turkey; ^2^ Department of Biostatistics Amasya University Faculty of Medicine Amasya Turkey; ^3^ Department of Internal Medicine Gebze Fatih State Hospital Kocaeli Turkey

**Keywords:** acute pancreatitis, albumin, biomarkers, disease severity, uric acid

## Abstract

**Aim:**

The uric acid‐to‐albumin ratio (UAR) has shown prognostic value in several inflammatory conditions, but its role in acute pancreatitis (AP) remains unclear. This study evaluated the prognostic value of UAR for disease severity in patients with AP.

**Methods:**

In this single‐center, retrospective study, 147 patients hospitalized with acute pancreatitis between February 2025 and June 2026 were included. UAR, systemic immune‐inflammation index (SII), and platelet‐to‐lymphocyte ratio (PLR) were calculated. Disease severity was classified using the revised Atlanta classification and the BISAP score. Diagnostic performance was assessed by receiver operating characteristic (ROC) analysis with pairwise area under the curve (AUC) comparisons (DeLong method).

**Results:**

Thirty‐eight patients (25.9%) had moderate‐to‐severe AP. UAR was higher in the moderate‐to‐severe group than in the mild group (1.65 ± 0.47 vs. 1.27 ± 0.34; *p* < 0.001) and increased across BISAP score categories (*p* < 0.001). UAR had an AUC of 0.741 (95% CI: 0.662–0.810), compared with 0.706 for SII and 0.646 for PLR; however, pairwise differences were not statistically significant. At a cut‐off of > 1.54 (Youden index), UAR had a specificity of 82.57% and a negative predictive value of 84.90%. Combining UAR with age increased the AUC to 0.801 and the negative predictive value to 88.57%. Bootstrap validation yielded an optimism‐corrected AUC of 0.794. UAR was associated with moderate‐to‐severe AP in univariable analysis (OR: 10.517; 95% CI: 3.770–29.336; *p* < 0.001), but not after multivariable adjustment (adjusted OR: 3.462; 95% CI: 0.870–13.773; *p* = 0.078).

**Conclusions:**

UAR is a simple, inexpensive biomarker with potential utility in acute pancreatitis severity assessment. Although not independently predictive, it may provide complementary information alongside clinical and laboratory findings.

## 1 Introduction

Acute pancreatitis is an inflammatory disease of the pancreas that develops in response to a variety of etiological factors. Although the clinical course is most often characterized by mild and self‐limiting forms, in some patients it may progress to a severe clinical picture accompanied by life‐threatening complications and organ failure [1]. Systemic organ failure, with or without local complications such as pancreatic necrosis and abscess, is one of the most important determinants of mortality in acute pancreatitis [2]. The most common etiological factors of acute pancreatitis are biliary disease and alcohol use, although medications, metabolic disorders, and procedural causes may also be involved [3]. In some patients, no underlying cause can be identified, and these cases are classified as idiopathic.

Patients most commonly present to emergency departments with abdominal pain, and clinical findings do not always parallel disease severity. The most widely used classification system in clinical practice is the Atlanta classification, which categorizes acute pancreatitis into three clinical groups: mild, moderate, and severe. However, particularly in distinguishing moderate from severe pancreatitis, a follow‐up period of at least 48 h is required to assess persistent organ failure. The BISAP score, another scoring system, has gained prominence in recent years due to its simplicity and early applicability, serving as an effective bedside scoring system for predicting mortality and severe disease risk at the time of admission [4].

Despite the availability of current clinical scoring and imaging methods, important limitations persist in the early and accurate prediction of disease severity in acute pancreatitis. Accordingly, in recent years, studies focusing on the prognostic value of biomarkers derived from laboratory parameters have been increasing in addition to existing scoring systems. In this context, parameters such as the platelet‐to‐lymphocyte ratio (PLR) and the systemic immune‐inflammation index (SII) have been shown to be associated with disease severity and prognosis [5].

The uric acid‐to‐albumin ratio (UAR) has been shown to be associated with mortality, morbidity, and disease prognosis in various conditions related to inflammation and endothelial dysfunction. In acute pancreatitis, oxidative stress is involved in the inflammatory and tissue injury response, while systemic inflammation, increased capillary permeability, and the negative acute‐phase response may contribute to decreased serum albumin levels. Given the close relationship of uric acid with oxidative and inflammatory pathways, an elevated UAR may reflect the combined oxidative and inflammatory burden associated with a more severe disease course. A significant association has been demonstrated between UAR and cardiovascular disease‐related mortality. In addition, beyond cardiovascular diseases, evidence is available to suggest that UAR may serve as a prognostic biomarker in serious clinical conditions such as renal failure and sepsis [6, 7]. Furthermore, current studies indicate that UAR may be an easily obtainable biomarker for predicting all‐cause mortality in the early period [8].

The limitations inherent in current clinical processes and classification systems underscore the need for predictive biomarkers that are applicable at the time of admission, inexpensive, and readily accessible. Although the prognostic value of inflammatory ratios such as SII and PLR has been demonstrated in acute pancreatitis, the role of UAR in this disease has not yet been addressed in the literature. The present study aims, for the first time, to systematically evaluate the diagnostic performance of UAR for the early prediction of disease severity in acute pancreatitis and to address this important gap in the literature by comparing this performance with that of SII and PLR. The findings of our study are expected to provide a practical contribution to clinical decision‐making processes, particularly in emergency departments and healthcare facilities with limited resources.

## 2 Materials and Methods

### 2.1 Study Design and Population

This study was designed as a single‐center, retrospective, observational investigation. Patients aged 18 years and older who were hospitalized and followed up with a diagnosis of acute pancreatitis (AP) between February 2025 and June 2026 were included in the study. The diagnosis of AP was established in accordance with the revised Atlanta criteria, requiring the fulfillment of at least two of the following three criteria: (i) abdominal pain consistent with acute pancreatitis (sudden‐onset, severe epigastric pain radiating to the back); (ii) elevation of serum amylase and/or lipase levels to at least three times the upper limit of normal; and (iii) imaging findings consistent with acute pancreatitis on computed tomography (CT), magnetic resonance imaging (MRI), or abdominal ultrasonography (US).

**FIGURE 1:**
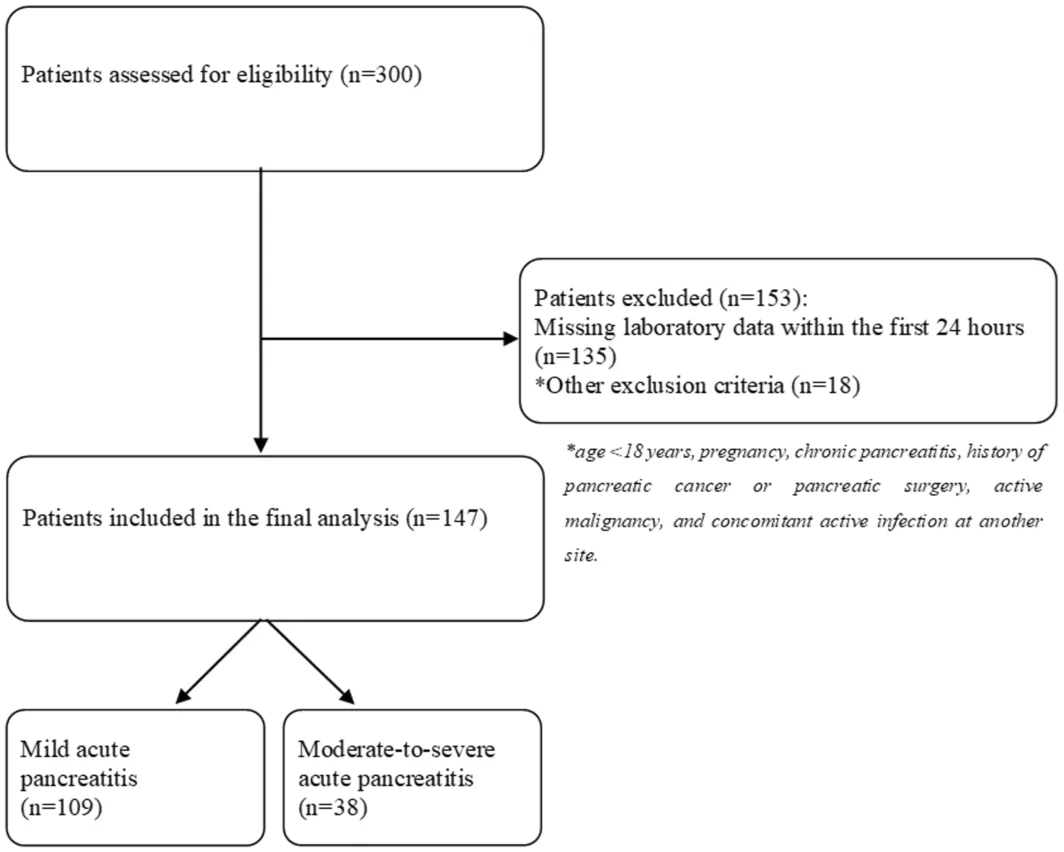
STROBE flow diagram of patient selection and classification according to acute pancreatitis severity.

The 300 patients were screened for the study, and patients who met one or more of the following exclusion criteria were excluded: (i) patients younger than 18 years of age; (ii) patients with a diagnosis of chronic pancreatitis; (iii) patients with a known history of pancreatic cancer or pancreatic surgery; (iv) patients with active malignancy at the time of admission; (v) pregnancy; (vi) patients in whom the uric acid‐to‐albumin ratio could not be calculated due to missing laboratory data; (vii) patients concurrently followed up with a diagnosis of an acute infectious disease other than acute pancreatitis. After the application of the exclusion criteria, a total of 147 patients were included in the study (Figure 1).

This study was conducted in accordance with the principles of the Declaration of Helsinki. Ethical approval was obtained from the Clinical Research Ethics Committee (approval date: 02/04/2026; approval number: 2026/002). Due to the retrospective design of the study, individual patient consent was not required.

### 2.2 Data Collection

Demographic, clinical, and laboratory data of the patients were obtained retrospectively from the electronic medical records of the hospital. Demographic data recorded at the time of admission included age and sex; clinical data included length of hospital stay (days) and mortality status (discharge/death).

Laboratory parameters were obtained from blood samples taken at the time of admission to the emergency department or within the first 24 h of hospitalization. The laboratory parameters analyzed were as follows: inflammatory markers (C‐reactive protein [CRP]); pancreatic enzymes (serum amylase, serum lipase); transaminases, bilirubins, and cholestatic enzymes (aspartate aminotransferase [AST], alanine aminotransferase [ALT], gamma‐glutamyl transferase [GGT], alkaline phosphatase [ALP], total bilirubin, direct bilirubin); hemogram parameters (white blood cell count [WBC], absolute neutrophil count, absolute lymphocyte count, hemoglobin, platelet count); and other biochemical parameters (serum uric acid, serum albumin).

### 2.3 Calculated Inflammatory Ratios

The following inflammatory ratios were calculated from the hemogram and biochemical parameters obtained at the time of admission:

#### 2.3.1 Uric Acid‐to‐Albumin Ratio (UAR)

Calculated by dividing the serum uric acid level (mg/dL) by the serum albumin level (g/dL).

#### 2.3.2 Platelet‐to‐Lymphocyte Ratio (PLR)

Calculated by dividing the platelet count by the absolute lymphocyte count.

#### 2.3.3 Systemic Immune‐Inflammation İndex (SII)

Calculated using the formula: platelet count × absolute neutrophil count/absolute lymphocyte count.

### 2.4 Classification of Disease Severity

The severity of acute pancreatitis was assessed using two different classification systems:

#### 2.4.1 Revised Atlanta Classification (2012)

Patients were divided into two groups: mild AP (no organ failure, no local or systemic complications) and moderate‐to‐severe AP (transient or persistent organ failure and/or local complications). The three categories defined in the original Atlanta classification (mild, moderate, severe) were combined into a binary (dichotomous) classification in order to prevent loss of statistical power due to the limited number of patients in the moderate and severe groups.

#### 2.4.2 BISAP (Bedside Index for Severity in Acute Pancreatitis) Score

Calculated within the first 24 h of admission using five parameters (BUN > 25 mg/dL, impaired mental status, presence of systemic inflammatory response syndrome [SIRS], age > 60 years, pleural effusion). Each parameter was assigned 1 point (total score: 0–5). Patients were stratified into three groups based on clinical relevance and previous studies: BISAP 0–1 (low risk), BISAP 2 (intermediate risk), and BISAP ≥ 3 (high risk).

### 2.5 Statistical Analysis

Statistical analyses were performed using IBM SPSS Statistics version 26.0 (IBM Corp., Armonk, NY, USA) and MedCalc Statistical Software version 20.0 (MedCalc Software Ltd., Ostend, Belgium). A *p*‐value < 0.05 was considered statistically significant for all tests.

#### 2.5.1 Descriptive Statistics

Continuous variables were presented as mean ± standard deviation (SD), and categorical variables as frequencies and percentages [*n* (%)].

#### 2.5.2 Assessment of Normality

The normality of continuous variables was assessed using the Kolmogorov–Smirnov test and histogram/*Q*–*Q* plots.

#### 2.5.3 Comparison of Two Independent Groups

Continuous variables between the two groups formed according to the Atlanta classification (mild AP and moderate‐to‐severe AP) were compared using the independent samples *t*‐test (Student's *t*‐test). The homogeneity of variances was assessed using Levene's test; when variances were not equal (Levene's test *p* < 0.05), the Welch‐corrected *t*‐test results were reported. For the comparison of categorical variables between groups, the chi‐square (*χ* ^2^) test was used, or Fisher's exact test was applied in cases where the expected cell count was less than 5.

#### 2.5.4 Comparison of Three İndependent Groups

One‐way analysis of variance (one‐way ANOVA) was used for the comparison of continuous variables among the BISAP score groups (0–1, 2, ≥ 3). Post hoc multiple comparison tests were performed for variables that showed significance in ANOVA. The chi‐square test was used for the comparison of categorical variables among the three groups.

#### 2.5.5 ROC Curve Analysis

The diagnostic performance of the calculated inflammatory ratios (UAR, PLR, SII) in discriminating moderate‐to‐severe AP (according to the Atlanta classification) was assessed using receiver operating characteristic (ROC) curve analysis. The area under the curve (AUC) and 95% confidence intervals (CI) were calculated. The statistical significance of AUC values compared with 0.5 (the reference line) was tested using the z‐statistic. Optimal cut‐off points were determined using the Youden index (J = sensitivity + specificity − 1), which simultaneously maximizes sensitivity and specificity. Differences in AUC values between ROC curves of different biomarkers were compared using the non‐parametric method proposed by DeLong et al. To assess the internal validity of the combined UAR + age model and account for potential overfitting inherent to a single‐center cohort, Harrell's bootstrap optimism‐correction procedure (2000 resamples) was applied and an optimism‐corrected AUC was calculated.

## 3 Results

### 3.1 General Characteristics of the Study Population

A total of 147 patients diagnosed with acute pancreatitis (AP) were included in the study. The mean age of the patients was 61.86 ± 17.13 years (range: 24–93). According to the Atlanta classification, 109 patients (74.1%) were classified as having mild AP and 38 (25.9%) as having moderate‐to‐severe AP. The distribution of BISAP scores was as follows: 96 patients (65.3%) had BISAP 0–1, 25 patients (17.0%) had BISAP 2, and 26 patients (17.7%) had BISAP ≥ 3.

### 3.2 Comparison According to the Atlanta Classification

When the mild AP group (*n* = 109) and the moderate‐to‐severe AP group (*n* = 38) were compared according to the Atlanta classification, the mean age of patients in the moderate‐to‐severe AP group (70.71 ± 13.87 years) was significantly higher than that of the mild AP group (58.77 ± 17.14 years) (*p* < 0.001). Length of hospital stay was also significantly longer in the moderate‐to‐severe AP group (8.81 ± 7.01 vs. 4.16 ± 1.54 days; *p* < 0.001). Mortality was observed only in the moderate‐to‐severe AP group (*n* = 2; 5.3%).

When inflammatory markers were evaluated, CRP levels were significantly higher in the moderate‐to‐severe AP group (58.84 ± 78.18 vs. 23.87 ± 32.94 mg/L; *p* < 0.001). In contrast, no statistically significant differences were observed between the groups in pancreatic enzyme levels, including amylase and lipase (*p* = 0.625 and *p* = 0.743, respectively). No significant differences were observed in the mean values of hepatic markers between the groups (all *p* > 0.05) (Table 1).

**TABLE 1:** Comparison of demographic, clinical characteristics, and laboratory parameters between the mild and moderate‐to‐severe acute pancreatitis groups according to the Atlanta classification.

Variable	Mild (*n* = 109)	Moderate‐to‐severe (*n* = 38)	*p*
Demographic and clinical characteristics			
Age (years)	58.77 ± 17.14	70.71 ± 13.87	**< 0.001**
Length of hospital stay (days)	4.16 ± 1.54	8.81 ± 7.01	**< 0.001**
Inflammatory markers			
CRP (mg/L)	23.87 ± 32.94	58.84 ± 78.18	**< 0.001**
Pancreatic enzymes			
Amylase (U/L)	1591.92 ± 1416.01	1720.84 ± 1342.57	0.625
Lipase (U/L)	4013.29 ± 4145.40	3763.97 ± 3599.33	0.743
Hepatic markers			
AST (U/L)	152.00 ± 177.39	171.87 ± 132.54	0.529
ALT (U/L)	126.92 ± 158.34	141.13 ± 139.84	0.625
GGT (U/L)	170.67 ± 196.65	204.42 ± 194.06	0.363
ALP (U/L)	111.73 ± 71.38	114.48 ± 64.97	0.856
Total bilirubin (mg/dL)	1.37 ± 1.87	1.94 ± 1.82	0.111
Direct bilirubin (mg/dL)	0.58 ± 1.16	0.96 ± 1.12	0.078

*Note:* Data are presented as mean ± standard deviation or *n* (%).

Abbreviations: ALP: alkaline phosphatase; ALT: alanine aminotransferase; AST: aspartate aminotransferase; CRP: C‐reactive protein; GGT: gamma‐glutamyl transferase.

When hemogram parameters were examined, the white blood cell count (WBC) (14.05 ± 5.35 vs. 10.73 ± 3.30 × 10^3^/μL; *p* < 0.001) and neutrophil count (12.02 ± 5.09 vs. 8.23 ± 3.21 × 10^3^/μL; *p* < 0.001) were significantly higher in the moderate‐to‐severe AP group, whereas the lymphocyte count was significantly lower (1.16 ± 0.72 vs. 1.66 ± 0.86 × 10^3^/μL; *p* = 0.002). No significant differences were observed between the groups regarding hemoglobin and platelet values. Among the biochemical parameters, the uric acid level was significantly higher in the moderate‐to‐severe AP group (5.36 ± 1.45 vs. 4.58 ± 1.18 mg/dL; *p* = 0.001), while the albumin level was significantly lower (3.32 ± 0.54 vs. 3.65 ± 0.39 g/dL; *p* < 0.001).

Marked differences between the groups were observed in terms of the calculated inflammatory ratios. The uric acid‐to‐albumin ratio (UAR) was approximately 1.3‐fold higher in the moderate‐to‐severe AP group compared to the mild AP group (1.65 ± 0.47 vs. 1.27 ± 0.34; *p* < 0.001). The platelet‐to‐lymphocyte ratio (PLR) (253.92 ± 151.18 vs. 189.95 ± 123.92; *p* = 0.011) and systemic immune‐inflammation index (SII) (3 162 551 ± 2 588 585 vs. 1.672 861 ± 1 508 319; *p* < 0.001) were also significantly higher in the moderate‐to‐severe AP group (Table 2).

**TABLE 2:** Comparison of hemogram parameters, biochemical parameters, and calculated inflammatory ratios according to the Atlanta classification.

Variable	Mild (*n* = 109)	Moderate‐to‐severe (*n* = 38)	*p*
Hemogram parameters			
WBC (×10^3^/μL)	10.73 ± 3.30	14.05 ± 5.35	**< 0.001**
Neutrophils (×10^3^/μL)	8.23 ± 3.21	12.02 ± 5.09	**< 0.001**
Lymphocytes (×10^3^/μL)	1.66 ± 0.86	1.16 ± 0.72	**0.002**
Hemoglobin (g/dL)	13.41 ± 1.74	13.24 ± 2.28	0.641
Platelets (×10^3^/μL)	241.84 ± 68.74	222.16 ± 87.77	0.160
Biochemical parameters			
Uric acid (mg/dL)	4.58 ± 1.18	5.36 ± 1.45	**0.001**
Albumin (g/dL)	3.65 ± 0.39	3.32 ± 0.54	**< 0.001**
Calculated inflammatory ratios			
Uric acid/Albumin ratio (UAR)	1.27 ± 0.34	1.65 ± 0.47	**< 0.001**
Platelet/Lymphocyte ratio (PLR)	189.95 ± 123.92	253.92 ± 151.18	**0.011**
Systemic Immune‐Inflammation Index (SII)	1.67 ± 1.50 × 10^6^	3.16 ± 2.58 × 10^6^	**< 0.001**

*Note:* Data are presented as mean ± standard deviation.

Abbreviations: PLR: platelet‐to‐lymphocyte ratio; SII: systemic immune‐inflammation index (platelets × neutrophils/lymphocytes); UAR: uric acid to‐albumin ratio; WBC: white blood cell count.

### 3.3 Comparison According to the BISAP Score

Comparisons among patients stratified into three groups according to the BISAP score (BISAP 0–1, BISAP 2, BISAP ≥ 3) using one‐way analysis of variance are presented in Table 3. The mean age increased significantly with increasing BISAP score (55.67 ± 16.16, 73.28 ± 12.83, and 73.73 ± 11.82 years, respectively; *F* = 23.68; *p* < 0.001). Length of hospital stay also increased with the score (4.10 → 5.92 → 9.52 days; *F* = 20.74; *p* < 0.001). Mortality was observed only in the BISAP ≥ 3 group (*n* = 2; 7.7%; *p* = 0.008).

**TABLE 3:** Comparison of demographic, clinical, and laboratory parameters according to the BISAP score groups.

Variable	BISAP 0–1 (*n* = 96)	BISAP 2 (*n* = 25)	BISAP ≥ 3 (*n* = 26)	*p*
Demographic and clinical characteristics				
Age (years)	55.67 ± 16.16	73.28 ± 12.83	73.73 ± 11.82	**< 0.001**
Length of hospital stay (days)	4.10 ± 1.75	5.92 ± 3.07	9.52 ± 7.95	**< 0.001**
Mortality, *n* (%)	0 (0.0)	0 (0.0)	2 (7.7)	**0.008**
Inflammatory markers				
CRP (mg/L)	27.00 ± 36.33	18.46 ± 41.82	68.62 ± 82.15	**< 0.001**
Pancreatic enzymes				
Amylase (U/L)	1512.46 ± 1419.68	1846.40 ± 1192.91	1829.04 ± 1477.33	0.407
Lipase (U/L)	3860.38 ± 3869.86	3686.36 ± 2988.66	4516.08 ± 5233.28	0.716
Hepatic markers				
AST (U/L)	124.54 ± 136.82	249.36 ± 193.79	188.81 ± 203.86	**0.002**
ALT (U/L)	113.59 ± 136.38	184.80 ± 169.49	141.23 ± 187.26	0.109
GGT (U/L)	175.73 ± 216.47	190.20 ± 111.07	182.73 ± 185.22	0.944
ALP (U/L)	103.60 ± 65.10	115.83 ± 67.35	136.77 ± 80.28	0.147
Total bilirubin (mg/dL)	1.26 ± 1.38	2.07 ± 2.97	1.94 ± 1.99	0.070
Direct bilirubin (mg/dL)	0.50 ± 0.86	1.06 ± 1.85	0.95 ± 1.17	**0.040**

*Note:* Data are presented as mean ± standard deviation or *n* (%). One‐way analysis of variance (ANOVA) was used. Statistically significant *p* values are shown in bold.

Among the laboratory parameters, the CRP level was significantly higher in the BISAP ≥ 3 group compared with the other two groups (68.62 ± 82.15 mg/L; *F* = 8.94; *p* < 0.001). AST levels differed significantly between the groups (*F* = 6.60; *p* = 0.002), and direct bilirubin values also reached significance (*F* = 3.29; *p* = 0.040). Amylase, lipase, ALT, GGT, and ALP levels did not differ significantly between the groups (all *p* > 0.05) (Table 3).

When hemogram parameters were evaluated, WBC and neutrophil counts were significantly higher in the BISAP ≥ 3 group compared with the other groups (*F* = 19.17 and *F* = 25.62, respectively; both *p* < 0.001). The lymphocyte count reached its lowest level in the BISAP 2 group (933 ± 423/μL), and the difference between the groups was significant (*F* = 14.78; *p* < 0.001). No significant differences were observed between the groups in terms of hemoglobin and platelet values.

Among the biochemical parameters, the uric acid level increased progressively with the BISAP score (4.45 → 4.70 → 6.13 mg/dL; *F* = 22.26; *p* < 0.001), and the albumin level reached its lowest value in the BISAP ≥ 3 group (3.19 ± 0.45 g/dL; *F* = 11.86; *p* < 0.001). The uric acid‐to‐albumin ratio (UAR) increased approximately 1.5‐fold with the BISAP score (1.23 → 1.33 → 1.92; *F* = 48.03; *p* < 0.001) and had the highest *F*‐statistic value among all parameters analyzed (Table 4).

**TABLE 4:** Comparison of hemogram parameters, biochemical parameters, and calculated inflammatory ratios according to the BISAP score groups.

Variable	BISAP 0–1 (*n* = 96)	BISAP 2 (*n* = 25)	BISAP ≥ 3 (*n* = 26)	*p*
Hemogram parameters				
WBC (×10^3^/μL)	10.58 ± 3.01	11.23 ± 4.53	15.67 ± 5.11	**< 0.001**
Neutrophils (×10^3^/μL)	7.94 ± 2.84	9.55 ± 4.15	13.56 ± 5.08	**< 0.001**
Lymphocytes (×10^3^/μL)	1.78 ± 0.86	0.93 ± 0.42	1.18 ± 0.74	**< 0.001**
Hemoglobin (g/dL)	13.51 ± 1.77	13.14 ± 1.89	13.04 ± 2.28	0.426
Platelets (×10^3^/μL)	244.31 ± 69.19	204.60 ± 52.36	239.77 ± 100.83	0.056
Biochemical parameters				
Uric acid (mg/dL)	4.45 ± 1.10	4.70 ± 1.02	6.13 ± 1.38	**< 0.001**
Albumin (g/dL)	3.65 ± 0.40	3.62 ± 0.49	3.19 ± 0.45	**< 0.001**
Calculated inflammatory ratios				
Uric acid/Albumin ratio (UAR)	1.23 ± 0.31	1.33 ± 0.35	1.92 ± 0.36	**< 0.001**
Platelet/Lymphocyte ratio (PLR)	170.24 ± 93.57	279.76 ± 181.51	269.84 ± 159.03	**< 0.001**
Systemic Immune‐Inflammation Index (SII)	1.41 ± 0.98 × 10^6^	2.78 ± 2.40 × 10^6^	3.75 ± 2.81 × 10^6^	**< 0.001**

*Note:* Data are presented as mean ± standard deviation. One‐way analysis of variance (ANOVA) was used. Statistically significant *p* values are shown in bold.

Abbreviations: PLR: platelet‐to‐lymphocyte ratio; SII: systemic immune‐inflammation index (platelets × neutrophils/lymphocytes); UAR: uric acid‐to‐albumin ratio; WBC: white blood cell count.

### 3.4 Logistic Regression Analyses

Univariable and multivariable logistic regression analyses were performed to identify factors associated with moderate‐to‐severe versus mild acute pancreatitis. In the univariable analyses, older age, longer hospital stay, higher CRP, leukocyte, and neutrophil levels, lower lymphocyte counts, and higher UAR, PLR, and SII values were significantly associated with moderate‐to‐severe acute pancreatitis.

In the multivariable analysis, increasing age remained independently associated with moderate‐to‐severe acute pancreatitis (OR: 1.049; 95% CI: 1.002–1.098; *p* = 0.041). Similarly, a longer hospital stay was independently associated with moderate‐to‐severe acute pancreatitis (OR: 1.703; 95% CI: 1.317–2.202; *p* < 0.001). Although UAR showed a strong association with moderate‐to‐severe acute pancreatitis in the univariable analysis (OR: 10.517; 95% CI: 3.770–29.336; *p* < 0.001), the association remained in the direction of greater disease severity but did not reach statistical significance in the multivariable analysis (OR: 3.462; 95% CI: 0.870–13.773; *p* = 0.078) (Table 5).

**TABLE 5:** Univariable and multivariable logistic regression analyses for moderate‐to‐severe acute pancreatitis according to the Atlanta classification.

Parameter	Univariable analyses				Multivariable analyses			
	OR	Lower	Upper	*p*	OR	Lower	Upper	*p*
Age (years)	1.050	1.022	1.078	< 0.001	1.049	1.002	1.098	0.041
Length of hospital stay (days)	1.788	1.400	2.283	< 0.001	1.703	1.317	2.202	< 0.001
CRP (mg/L)	1.013	1.005	1.020	0.002	1.008	0.994	1.021	0.257
WBC (×10^3^/μL)	1.212	1.099	1.335	< 0.001	1.273	0.233	6.965	0.781
Neutrophil (×10^3^/μL)	1.259	1.134	1.398	< 0.001	0.859	0.144	5.107	0.867
Lymphocyte (×10^3^/μL)	0.419	0.237	0.740	0.003	0.249	0.022	2.838	0.263
UAR	10.517	3.770	29.336	< 0.001	3.462	0.870	13.773	0.078
PLR	1.003	1.001	1.006	0.017	0.996	0.982	1.010	0.529
SII	1.038	1.016	1.060	< 0.001	1.010	0.907	1.126	0.853

Abbreviations: CRP, C‐reactive protein; OR, odds ratio; PLR, platelet‐to‐lymphocyte ratio; SII, systemic immune‐inflammation index; UAR, uric acid‐to‐albumin ratio; WBC, white blood cell count.

### 3.5 ROC Curve Analysis

Receiver operating characteristic (ROC) curve analysis was performed to evaluate the diagnostic performance of the uric acid‐to‐albumin ratio (UAR) in discriminating moderate‐to‐severe acute pancreatitis. The area under the curve (AUC) for UAR was 0.741 (95% CI: 0.662–0.810; *p* < 0.0001). The optimal cut‐off value determined using the Youden index was > 1.54, at which sensitivity was 57.89% and specificity was 82.57%.

**FIGURE 2:**
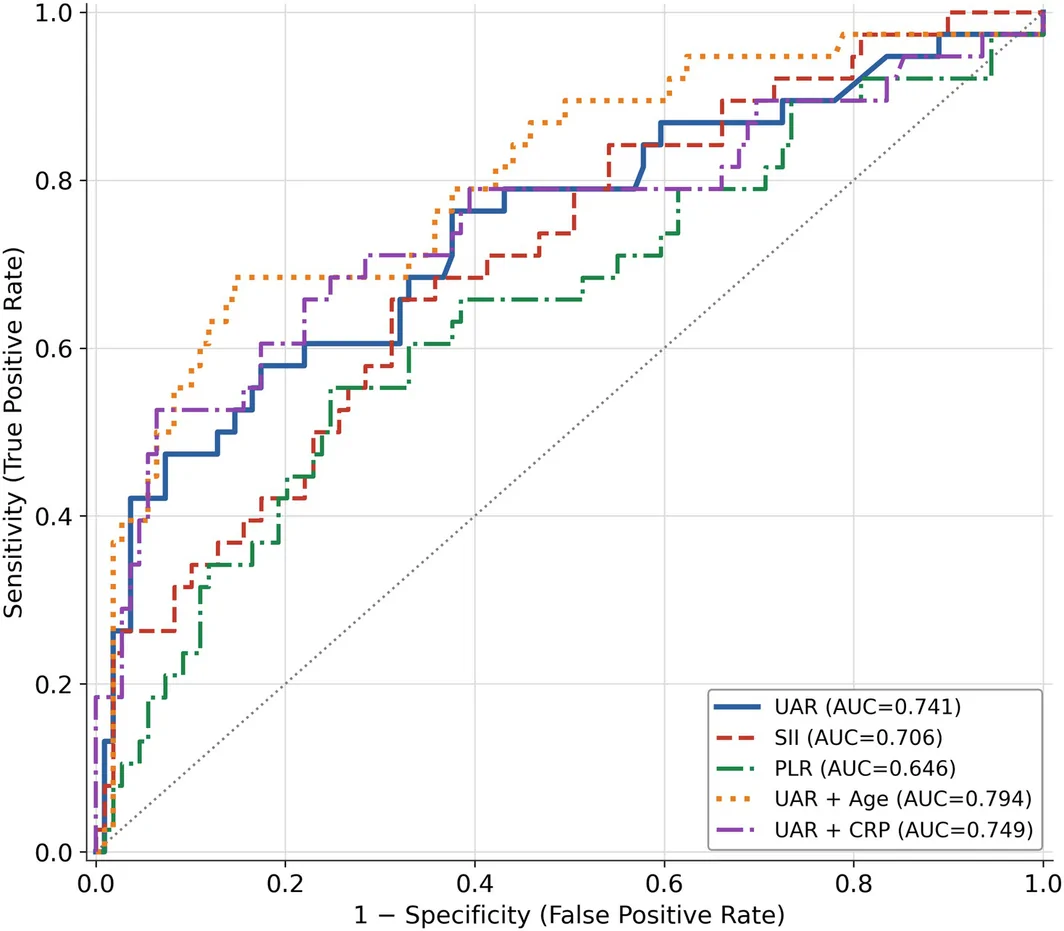
Receiver operating characteristic curves for predicting clinical severity in patients with acute pancreatitis.

When the diagnostic performance of UAR was compared with the other calculated inflammatory ratios, UAR had the numerically highest AUC value (AUC: 0.741). SII (AUC: 0.706; 95% CI: 0.625–0.778) and PLR (AUC: 0.646; 95% CI: 0.563–0.723) had lower AUC values. However, in pairwise ROC curve comparisons using the DeLong method, the differences between UAR and SII (AUC = 0.035; *p* = 0.604) and between UAR and PLR (AUC = 0.096; *p* = 0.221) did not reach statistical significance. Similarly, the difference between SII and PLR was not significant (AUC = 0.060; *p* = 0.077) (Table 6, Figure 2). Combining UAR with age increased the AUC to 0.801 (95% CI: 0.728–0.863); internal validation using Harrell's bootstrap optimism‐correction method (2000 resamples) yielded a corrected AUC of 0.794, with minimal optimism (0.005), supporting the internal robustness of this combined model within the present cohort.

**TABLE 6:** ROC analysis results of UAR and combined models for the prediction of moderate‐to‐severe acute pancreatitis.

Parameter	Cut‐off	AUC (95% CI)	*p*	Sens. (%)	Spec. (%)	PPV (%)	NPV (%)	Youden index	Accuracy (%)
UAR	> 1.54	0.741 (0.662–0.810)	< 0.001	57.89	82.57	53.66	84.90	0.405	76.19
SII	1.76 × 10^6^	0.706 (0.625–0.778)	< 0.001	65.79	68.81	42.38	85.23	0.346	68.03
PLR	218	0.646 (0.563–0.723)	0.008	55.26	75.23	43.75	82.83	0.305	70.07
UAR + Age	> 0.32	0.794 (0.728–0.863)	< 0.001	68.42	85.32	61.90	88.57	0.537	80.95
UAR + CRP	> 0.41	0.749 (0.671–0.817)	< 0.001	52.63	93.58	74.08	85.00	0.462	82.99

Abbreviations: AUC: area under the curve; CI: confidence interval; CRP: C‐reactive protein; NPV: negative predictive value; PLR: platelet‐to‐lymphocyte ratio; PPV: positive predictive value; Sens.: sensitivity; SII: systemic immune‐inflammation index; Spec.: specificity; UAR: uric acid‐to‐albumin ratio.

## 4 Discussion

To our knowledge the present study is one of the first clinical investigations to evaluate the relationship between the uric acid‐to‐albumin ratio (UAR) and disease severity in acute pancreatitis (AP). Acute pancreatitis is a disease with a broad clinical spectrum; although the majority of mild cases are self‐limiting, mortality in moderate‐to‐severe forms may reach 20%–30% [9]. Although the revised Atlanta classification remains the gold standard, the requirement of a 48‐h observation period for the assessment of persistent organ failure limits early risk stratification at the time of admission [10]. The BISAP score, although applicable in the early period, is insufficient on its own to reflect the entire clinical spectrum of the disease. This clinical gap highlights the need for rapid, inexpensive, and universally accessible biomarkers.

In our study, according to the Atlanta classification, 74.1% of the patients were classified as having mild AP and 25.9% as having moderate‐to‐severe AP. According to the BISAP classification, 65.3% of the patients scored 0–1, 17.0% scored 2, and 17.7% scored ≥ 3. This distribution is consistent with that reported in the current literature [11, 12]. The distribution of our study population across the clinical severity categories was broadly consistent with previous reports, supporting the representativeness of our cohort.

Age was significantly associated with disease severity in both classification systems. Similarly, in a study by Akca et al., patients older than 75 years had significantly higher rates of severe disease [12].

Our findings indicate that CRP, white blood cell count, neutrophil and lymphocyte counts, as well as uric acid and albumin levels are significant laboratory markers reflecting disease severity according to both the Atlanta and BISAP classifications. These findings are consistent with the existing literature demonstrating an association between inflammatory markers and disease severity in acute pancreatitis [5].

Although UAR has been shown in the current literature to have prognostic value in sepsis, acute kidney injury, and cardiovascular diseases [6, 7, 8, 13, 14, 15, 16, 17], this gap has not been previously addressed in the context of acute pancreatitis. Our findings suggest that UAR is significantly and progressively associated with disease severity according to both the Atlanta classification and the BISAP score, supporting its potential role as a candidate biomarker for severity assessment in acute pancreatitis.

When the three inflammatory ratios evaluated in our study were compared, UAR yielded the numerically highest AUC value (AUC = 0.741), followed by SII (AUC = 0.706) and PLR (AUC = 0.646). However, pairwise comparisons using the DeLong test did not demonstrate statistically significant differences between these markers; therefore, these findings should not be interpreted as evidence of superior discriminative performance of UAR. As emphasized in a recent study, the clinical value of a novel biomarker should ideally be supported by demonstrable statistical superiority over existing markers or by retained independent significance in multivariable models [18]; UAR met neither criterion in the present cohort, a limitation that should be weighed when interpreting its clinical relevance. At the selected cut‐off, UAR showed a specificity of 82.57% and a negative predictive value of 84.90%, suggesting that it may contribute to early risk assessment. Nevertheless, its modest sensitivity limits its use as a stand‐alone rule‐out tool. These findings are consistent with previous studies reporting the prognostic value of SII and PLR in acute pancreatitis [19, 20, 21]. Although UAR was significantly associated with moderate‐to‐severe acute pancreatitis in the univariable analysis, this association did not remain statistically significant in the multivariable analysis. This finding may be attributable to the limited number of patients with moderate‐to‐severe acute pancreatitis in our cohort and to the inclusion of inflammatory variables that reflect overlapping biological processes in the model. Therefore, although our findings do not confirm UAR as an independent predictor, they suggest that it may serve as a complementary biomarker within multiparametric risk assessment models.

From a clinical standpoint, a notable finding was the numerically higher AUC of 0.801 and NPV of 88.57% when UAR was combined with age. These findings suggest that, rather than being used as a stand‐alone biomarker, UAR may have potential as a complementary component of early risk assessment models. Internal bootstrap validation of this combined model (2000 resamples) yielded minimal optimism (corrected AUC = 0.794), suggesting limited overfitting within this cohort; nonetheless, given the modest sample size and single‐center design, external validation in independent, multicenter cohorts remains necessary before clinical application. The increase in the AUC value to 0.749 when UAR was combined with CRP also suggests that this model may have a complementary role in identifying high‐risk patients. Recent data from Zhou et al., demonstrating that the combined use of inflammation‐based biomarkers with the SOFA score enhances prognostic performance, parallels and supports our findings [22]. The fact that UAR can be calculated from routine biochemical panels without additional cost provides a clear advantage in terms of applicability, particularly in regional hospitals and emergency departments where advanced laboratory facilities are limited.

The strengths of our study include the concurrent use of two different clinical severity classification systems (Atlanta and BISAP), the comparative analysis of three inflammatory ratios, and the evaluation of combined models. Nevertheless, several limitations should be considered: (i) the single‐center and retrospective design may have introduced selection bias; (ii) the number of patients in the moderate‐to‐severe AP group (*n* = 38) was below the minimum threshold suggested by post hoc power analysis; (iii) the low mortality rate (1.4%) precluded a separate evaluation of the role of UAR in predicting mortality; and (iv) since only values obtained at the time of admission were used, the kinetics of the biomarkers could not be analyzed.

## 5 Conclusions

UAR is a low‐cost and readily available biomarker derived from routine biochemical parameters. In our study, UAR showed a significant univariate association with moderate‐to‐severe acute pancreatitis and discriminative performance comparable to that of SII and PLR, although its independent association was not confirmed in the multivariable analysis. The addition of age yielded an AUC of 0.801, with an optimism‐corrected AUC of 0.794 after bootstrap validation. Despite these limitations, the present study provides clinically relevant preliminary evidence regarding the potential role of UAR in acute pancreatitis and highlights its practical value as an inexpensive and easily accessible marker that may complement existing approaches to early severity assessment. These findings may serve as a basis for future prospective, multicenter studies designed to validate its prognostic utility and determine its place within integrated risk‐stratification models.

## Funding

The authors have nothing to report.

## Ethics Statement

Due to the retrospective nature of the study, the requirement for informed consent was waived by the local ethics committee. Approval was granted by the Clinical Research Ethics Committee of Amasya University (approval date: 02/04/2026; approval number: 2026/002).

## Conflicts of Interest

The authors declare no conflicts of interest.

## Data Availability

The datasets generated and/or analyzed during the current study are available from the corresponding author on reasonable request.
